# Exposure to alcohol and overall survival in head and neck cancer: A regional cohort study

**DOI:** 10.1002/hed.27125

**Published:** 2022-06-17

**Authors:** Alexander Denissoff, Teemu Huusko, Sami Ventelä, Solja Niemelä, Johannes Routila

**Affiliations:** ^1^ Department of Psychiatry, Faculty of Medicine University of Turku Turku Finland; ^2^ Addiction Psychiatry Unit, Department of Psychiatry Turku University Hospital Turku Finland; ^3^ Department for Otorhinolaryngology – Head and Neck Surgery University of Turku Turku Finland; ^4^ Department for Otorhinolaryngology – Head and Neck Surgery Turku University Hospital Turku Finland

**Keywords:** alcohol, history, HNSCC, survival, tobacco

## Abstract

**Background:**

There is a paucity of knowledge regarding the association of alcohol use with overall survival (OS) of patients with head and neck squamous cell carcinoma (HNSCC).

**Methods:**

All 1033 patients treated for new HNSCC in Southwest Finland regional referral center of Turku University Hospital in 2005–2015. Cox regression analysis was used. Tumor TNM classification, age at baseline and tobacco smoking status were assessed as potential confounders.

**Results:**

A history of severe harmful alcohol use with major somatic complications (HR: 1.41; 95%CI: 1.06–1.87; *p* = 0.017) as well as current use of at least 10 units per week (HR: 1.44, 95%CI: 1.16–1.78; *p* = 0.001) were associated with OS.

**Conclusions:**

Alcohol consumption of 10–20 units/week, often regarded as moderate use, was found to increase risk of mortality independent of other prognostic variables. Systematic screening of risk level alcohol use and prognostic evaluation of alcohol brief intervention strategies is highly recommended.

## 1 INTRODUCTION

Alcohol use is a well‐established risk factor for head and neck squamous cell carcinoma (HNSCC).^1^, ^2^, ^3^, ^4^ However, there is a paucity of knowledge regarding the association of alcohol use with prognosis of patients with HNSCC. In previous studies, concurrent alcohol use has been associated with treatment complication in surgery^5^ and chemotherapy,^6^ poorer outcomes in radiotherapy,^7^, ^8^ and longer hospitalizations.^5^ Despite this, a significant proportion of patients continues their prediagnosis alcohol consumption habits,^9^ which further underscores the importance of this issue.

Several high‐quality cohort studies have explored the association between alcohol use and overall survival in unselected patients with HNSCC.^10^, ^11^, ^12^, ^13^, ^14^, ^15^, ^16^, ^17^, ^18^ However, the studies are markedly heterogeneous in terms of sample characteristics and exposure variables utilized. Self‐report measures utilizing qualitative categories regarding character of use^12^ or proxy measures for alcohol use disorder^17^ have been used without providing detailed information regarding heaviness of consumption. Furthermore, some studies have been prospective in design, introducing the possibility of inclusion bias.^12^ Thus, the reported conclusions have been equivocal with some studies reporting significant positive findings in unstratified analyses.^10^, ^11^, ^12^ Other studies have resulted in positive findings only in analyses stratified by variables such as cancer site.^14^, ^15^, ^16^

The primary aim of our study is to examine the associations between problem level alcohol use and overall survival (OS) of patients with HNSCC utilizing multivariable statistical modeling and discriminating between current and former use. Extensive information on alcohol and tobacco use as well as other potential confounders allows for assessment of prognostic issues in a robust analytical framework. As the regional referral center is responsible for treatment of all HNSCC cases in Southwest Finland per national treatment guidelines, the study is void of inclusion bias induced by socioeconomic or insurance status‐related issues.^19^

## 2 METHODS

### 2.1 Participants and data‐collection

**FIGURE 1:**
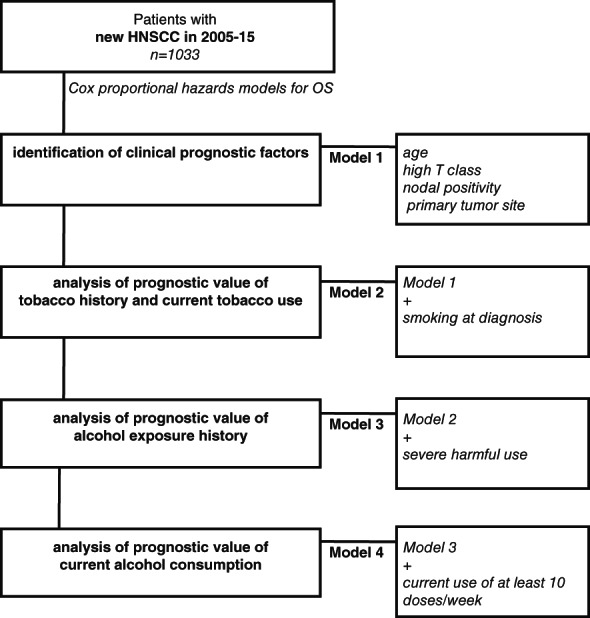
The study protocol. Including all 1033 novel HNSCC cases in Southwestern Finland region in 2005–2010, clinical prognostic factors, tobacco exposure and alcohol‐related variables were serially analyzed to form the four main statistical models of the study

The study protocol is shown in Figure 1. The HNSCC patient cohort included all patients treated for new HNSCC in Southwest Finland regional referral center of Turku University Hospital (TUH) in 2005–2015. Patients were identified using an electronic database screen, and the cohort inclusion verified by accessing the individual electronic patient charts. The charts were reviewed and pertinent clinicopathological data recorded. The study was approved by the Finnish national authority for medicolegal affairs (V/39706/2019), regional ethics committee of University of Turku (51/1803/2017). The authors assert that all procedures contributing to this work comply with the ethical standards of the relevant national and institutional committees on human experimentation and with the Helsinki Declaration of 1975, as revised in 2008.

### 2.2 Exposure variable: Alcohol use

Information on alcohol use was collected from the patient records. Alcohol use was estimated at the time of diagnosis by clinical interview to determine alcohol intake in units per week, and repeatedly at each visit. This is an established practice in the TUH clinic responsible for treating HNSCC cases. Current use was categorized into three groups of consuming less than 10 units per week, 10–20 per week or more than 20 units per week. On the first visit, the patients were asked to consider alcohol use in the last 6 months prior to diagnosis of HNSCC. A unit is defined as 12 g of pure alcohol.

Cumulative alcohol exposure was ascertained dichotomously as presenting with a history of problem level alcohol use (yes/no) defined as previous alcohol consumption more than 10 units/week. In addition, a history of severe alcohol‐related somatic complications was defined as the presence of alcohol‐related ICD‐10 diagnoses F10.31, F10.4, F10.40, F10.41, F10.6, F10.73, F10.01, F10.02, F10.06, F10.74, F10.6, G31.2, G40.51, G62.1, G72.1, K70*, I42.6, K85.2, K86.00, K86.01, K86.08, T510, T510* X45 or T510*X69, which include pancreatitis, alcohol hepatitis, alcohol polyneuropathy, or alcohol encephalopathy, in the medical records of the TUH.

### 2.3 Outcome variable: Overall survival

Overall survival was defined from end‐of‐treatment to end‐of‐follow‐up or death. Five‐year survival was preferentially used, and cause of death determined by chart review. Survivalship status was recorded from medical records of the TUH which have linkage to the Finnish National Population Information System database. Patients lost to follow‐up were censored at the last data in the hospital records.

### 2.4 Covariates

Tumors were staged according to TNM criteria applicable at the time of diagnosis. Treatment protocols followed decisions by the multidisciplinary Tumor Board for head and neck cancer.

Information on tobacco use was collected from the patient records. Tobacco history was determined at the time of diagnosis by clinical questions to determine tobacco use duration in years and the number of daily cigarette equivalents. On further visits, tobacco use was repeatedly inquired. Subjects were categorized by tobacco use status into four group: patients with no tobacco use for at least 5 years, patients who quitted tobacco use within 5 years, patients who quitted tobacco use at diagnosis, and patients with continued tobacco use after diagnosis of HNSCC. Cumulative tobacco use was estimated as pack‐years (PY), the product of the number of packs of cigarette equivalents smoked per day and the number of years of tobacco use. When other tobacco products than cigarettes were used, established conversion criteria were applied. In this study, subjects were categorized by PY years categories of never, PY < 10, PY < 20, PY < 30, PY < 40, and PY ≥ 40.

### 2.5 Statistical analyses

Statistical analyses were performed using SPSS statistical software (IBM SPSS Statistics, version 25; IBM Co., Armonk, NY). First, we applied multivariable Cox proportional hazards method to identify a preliminary prognostic model using backward stepwise regression using 5‐year OS, the likelihood ratio method, and exclusion *p*‐value of 0.10. All covariates presented in Table 1 were included. After identification of the preliminary prognostic Model 1 (patient age, high T classification, nodal positivity, and primary tumor site), the most robust tobacco‐related variable was identified using Cox proportional hazards method entering the preliminary prognostic model variables.

**TABLE 1:** The distributions and survival effects of the clinicopathological variables adjusted in a multivariable model

	Total		Survival at 5 years		Survival effect	
	No. of patients	%	No. of patients	%	HR (95%CI)	*p*‐value
Sex						
Male	679	66	369	54	Not included	
Female	354	34	200	56	Not included	
Age at diagnosis						
<65	487	47	292	60	1.04 (1.0–1.05)/year	<0.001
>65	546	53	277	51		
Primary tumor site						
Oral cavity	505	49	309	61	1	
Oropharynx	193	19	105	54	1.06 (0.81–1.38)	0.70
Larynx	184	18	98	53	1.21 (0.93–1.57)	0.15
Hypopharynx	40	4	5	13	2.99 (2.07–4.31)	<0.001
Other	111	11	52	47	1.25 (0.93–1.68)	0.14
T class						
T0‐2	676	65	466	69	0.30 (0.25–0.36)	<0.001
T3‐4	357	35	103	29	1	
N class						
N0	638	62	395	62	0.60 (0.49–0.74)	<0.001
N+	395	38	174	44	1	
Stage						
0‐II	481	47	345	72	Not included	
III‐IV	552	53	224	41	Not included	

*Note*: Survival effect was analyzed using a multivariable Cox proportional hazards model. Results of the final prognostic model are presented, including hazard ratios (HR), 95% confidence intervals (CI), and *p*‐values.

Next, the association of current and former alcohol use with OS was studied using Model 2 (patient age, high T classification, nodal positivity, primary tumor site, tobacco use at the time of diagnosis). The alcohol use history variables (i.e., problem level alcohol use and history of severe harmful use with major somatic complications) were included in Model 3, while the final prognostic Model 4 included current alcohol consumption as a variable (Model 4: patient age, high T classification, nodal positivity, primary tumor site, tobacco use at the time of diagnosis, history of severe harmful alcohol use with major somatic complications, current alcohol consumption of at least 10 units per week).

For all reported models, the proportionality of hazards was confirmed using log‐minus‐log plotting and plotting Schoenfeld residuals against survival time, when appropriate. For all Cox models, hazard ratios (HR) with 95% confidence intervals (CI), and *p*‐values are reported.

## 3 RESULTS

### 3.1 Preliminary prognostic model

A total of 1033 individuals with new primary HNSCC during the years 2005–2015 were identified and included in the cohort, the characteristics of which are shown in Table 1. Most cancers were non‐metastasized and of limited extent. Oral cavity was the most common subsite. In all, 569 patients were alive after 5 years of follow‐up, while 316 patients died of HNSCC and 148 patients of other causes. The 5‐year overall survival (OS) was thus 55% and disease‐specific survival 69%. Surgery was included in the treatment plan of 734 patients (71%) and oncological therapy was used in the treatment of 616 patients (60%). Survival was strongly impacted by age, high T classification, nodal positivity, and primary tumor site (Table 1).

### 3.2 Current smoking is a strong predictor of OS

Cohort characteristics in terms of cumulative and current tobacco use are presented in Table 2. Using a multivariable model adjusting for the identified clinical prognostic variables (age, high T classification, nodal positivity, and primary tumor site), the prognostic significance of cumulative tobacco use was evaluated (Table 2). Testing for a linear trend over the cumulative tobacco use history variable indicative of dose–response, each 10 PY increase in tobacco history was associated with a HR of 1.09 (95%CI: 1.03–1.16, *p* = 0.004). Using different PY cut‐offs, both exposure history of at least 20 PY (HR: 1.28; 95%CI: 1.04–1.58; *p* = 0.021) as well as exposure history of at least 40 PY (HR: 1.37; 95%CI: 1.18–1.68; *p* = 0.002) associated with OS (Figure S1A,B, Supporting Information).

**TABLE 2:** The survival effects of history of tobacco use and tobacco use at the time of treatment

	Total		Survival at 5 years		Survival effect	
	No. of patients	%	No. of patients	%	HR (95%CI)	*p*‐value
Tobacco history						
Less than 10 pack years	408	39	245	60	1	
10–20 pack years	75	7	47	63	1.01 (0.67–152)	0.96
20–30 pack years	96	9	58	60	1.06 (0.73–1.53)	0.77
30–40 pack years	132	13	77	58	1.12 (0.81–1.55)	0.48
Over 40 pack years	322	31	142	44	1.42 (1.17–1.81)	0.004
Tobacco use at the time of treatment						
No tobacco use for at least 5 years	535	52	320	60	1	
Quitted within 5 years	35	3	25	71	0.76 (0.40–1.45)	0.41
Quitted at diagnosis	134	13	76	57	1.30 (0.95–1.79)	0.11
Continued tobacco use	329	32	148	45	1.77 (1.40–2.24)	<0.001

*Note*: Survival effect was analyzed using a multivariable Cox proportional hazards model adjusting for patient age, high T class, nodal positivity, and primary tumor site. Hazard ratios (HR), 95% confidence intervals (CI), and *p*‐values are presented.

However, in prognostic models adjusting for current tobacco use, the cumulative tobacco exposure variables were found to lose their OS impact. Thus, the impact of current tobacco use was carefully evaluated (Table 2). Continued tobacco use after diagnosis was expectedly associated with a poor prognosis (Figure S1C) (HR: 1.67; 95%CI: 1.36–2.05; *p* < 0.001). However, quitting tobacco use at diagnosis did not result in an unequivocal improvement in prognosis. Thus, tobacco use at the time of diagnosis was revealed to be the strongest predictor of OS (Figure S1D) (HR: 1.67; 95%CI: 1.34–2.07; *p* < 0.001) and was accordingly included in the final prognostic model.

### 3.3 Alcohol use is a strong predictor of OS

**FIGURE 2:**
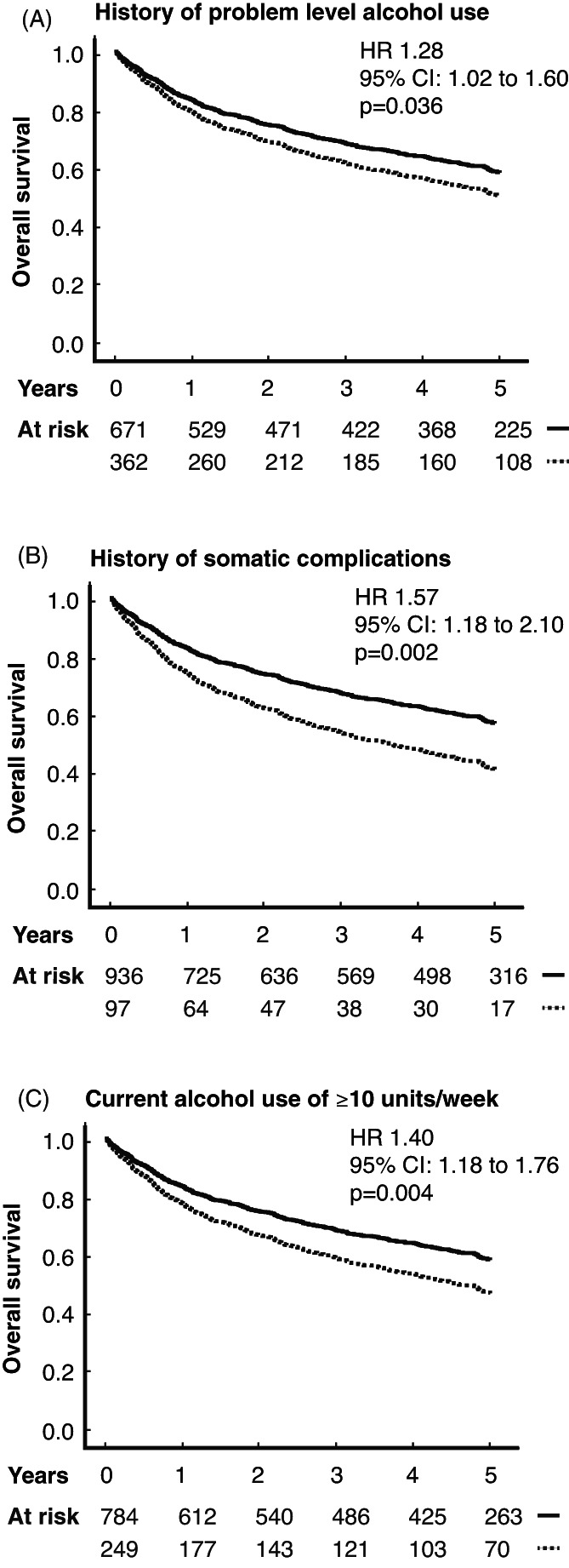
The relationship between alcohol exposure and overall survival (OS). History of (A) problem level alcohol use and (B) severe harmful alcohol use with major somatic complications were strongly associated with OS. (C) In the final statistical model, current alcohol consumption of at least 10 units/week remained a highly significant prognostic variable. CI, confidence interval; HR, hazard ratio

Cohort characteristics in terms of current and former alcohol exposure are presented in Table 3. Using a multivariable model adjusting for the identified clinical prognostic variables (age, high T classification, nodal positivity, and primary tumor site) and current tobacco use at the time of diagnosis, the prognostic impact of the alcohol exposure history was assessed (Table 3). History of problem level alcohol use was associated with poor survival (Figure 2A) (HR: 1.28; 95%CI: 1.02–1.60; *p* = 0.036). Even more strongly, however, history of severe alcohol‐related somatic complications such as pancreatitis, alcohol hepatitis, or alcohol encephalopathy (cf. section 2) was found to associate with poor survival (Figure 2B) (HR: 1.57; 95%CI: 1.18–2.10; *p* = 0.002).

**TABLE 3:** The survival effects of alcohol use history and current alcohol use

	Total		Survival at 5 years		Survival effect	
	No. of patients	%	No. of patients	%	HR (95%CI)	*p*‐value
Alcohol use history						
No alcohol history	671	65	399	59	1	
Previous problem use	265	26	136	51	1.16 (0.91–1.49)	0.23
Somatic complications	97	9	34	35	1.71 (1.24–2.35)	0.001
Current alcohol use						
Less than 10 doses/week	784	76	461	59	1	
10–20 doses per week	122	12	52	43	1.54 (1.16–2.03)	0.002
Over 20 doses/week	127	12	56	44	1.33 (1.00–1.77)	0.050

*Note*: Survival effect was analyzed using a multivariable Cox proportional hazards model adjusting for patient age, high T class, nodal positivity, primary tumor site, and smoking at the time of diagnosis. Hazard ratios (HR), 95% confidence intervals (CI), and *p*‐values are presented.

The survival impact of current alcohol use was investigated in a multivariable model, where patient age, high T classification, nodal positivity, primary tumor site, tobacco use at the time of diagnosis, and history of severe alcohol‐related somatic complications were entered as covariates. Current problem level alcohol use was associated with a poor prognosis (Figure 2C) (HR: 1.40; 95%CI: 1.18–1.76; *p* = 0.004). The survival impact of severe alcohol history remained significant in the final model. No significant interactions were found between current alcohol use, current tobacco use, cumulative tobacco use history, and history of alcohol use.

### 3.4 Treatment effects

Finally, the confounder bias introduced by differences in the treatment protocols was evaluated (Table S1). When the treatment variations were assessed using the final Model 4, patients with no treatment and patients treated with radiotherapy alone had a significantly worse survival. Importantly however, current problem‐level alcohol use was a significant prognostic in radiotherapy‐treated patients (HR: 1.65; 95%CI: 1.27–2.15; *p* < 0.001).

## 4 DISCUSSION

This large population‐based regional cohort study of patients with HNSCC reports a strong association between current problem level alcohol use, history of severe alcohol‐related somatic complications and 5‐year OS. Importantly, current alcohol use retained its prognostic worth despite adjustment for history of severe alcohol‐related somatic complications and previously identified clinical prognostic variables.^19^ Of all the studies addressing this issue, ours is the first one to utilize a cohort encompassing all incident HNSCC patients in a regional population.

The previously published cohort studies reporting a significant positive finding in unstratified analyses^10^, ^11^, ^12^ have been heterogeneous in terms of exposure variables as well as sample sizes and characteristics. Importantly, prospective settings introduce the possibility of selection bias. Qualitative self‐report measures for alcohol use have lacked information on amount of units consumed/week.^12^ In contrast, other investigations have utilized alcohol use variables categorized by grams consumed per day (abstainer, <23, 23–46, or >46 g), which preclude from evaluating moderately heavy consumption‐related outcomes.^11^ Studies reporting large effect sizes have suffered from lack of control for confounders such as tobacco use.^10^

In our sample, history of problem level alcohol use was also associated with impaired survival. The association was even stronger for a history of severe alcohol‐related somatic complications, possibly due to an independent adverse prognostic influence of alcohol‐related somatic diseases. The lack of linearity between survival impacts of current exposure levels is most likely due to bias introduced by the adjustment for complicated alcohol use history as well as the greater likelihood of interventions when severe alcohol use is reported. Most importantly, moderately heavy consumption (10–20 units/week) was associated with OS. This is of special clinical significance as this group includes patients with problem level alcohol use but not necessarily meeting criteria for an alcohol use disorder (AUD).

Concerns have been raised regarding underscreening and non‐treatment of problem level alcohol use in patients with HNSCC.^20^ Most importantly, persons with risk‐level alcohol use may benefit from screening and brief intervention (SBI) implemented systematically alongside HNSCC treatment and follow‐up. SBI typically includes systematical screening of hazardous alcohol use, for example, using AUDIT‐questionnaire in a clinical context and giving feedback on alcohol use utilizing motivational interviewing approach.^21^, ^22^ Meta‐analytic evidence indicates that 1 in 10 of patients with hazardous alcohol use receiving SBI convert to non‐hazardous use or abstinence.^23^, ^24^ On the other hand, SBI is not efficacious in people with severe AUD or very heavy drinking.^22^, ^23^, ^24^ Particularly, it should be noted that cutting down alcohol consumption reduces mortality and burden of disease on the population level and thus is to be regarded as a meaningful treatment goal as opposed to solely focusing on achieving abstinence.^25^ Regrettably, only one randomized controlled trial of a treatment intervention for hazardous/harmful alcohol use in patients with HNSCC^26^ was identified in a recent systematic review.^27^ There, a negative finding was reported. However, problem drinking was defined in this trial by an Alcohol Use Disorders Identification Test (AUDIT) cutoff score of 8 with no information provided on heaviness of use as measured by units of alcohol or grams of ethanol consumed per week. This is a crucial issue, as other studies assessing the efficacy of SBI in inpatient settings have utilized samples comprising of heavy drinkers and thus reported negative results.^28^, ^29^ Furthermore, as patients with cancer are especially prone to participate in lifestyle interventions,^30^ it is plausible that moderately heavily drinking patients with HNSCC are capable of benefiting from SBI.

The strengths of this study are as follows: The main strength of this study is the large sample size, which included all patients with HNSCC within a 10‐year period treated at our regional referral center. As all diagnosed cases of HNSCC are referred to our tertiary referral center from the complete Southwestern Finland region per national treatment guidelines, the study is void of bias inflicted by socioeconomic or insurance status. Moreover, although Finnish alcohol consumption levels per capita do not markedly differ from those other European countries,^31^ problem drinking is particularly ubiquitous in Finland.^32^ Thus, providing a regional unselected sample, our study is well poised to assess hypotheses concerning prognostic issues regarding especially problem level alcohol use. Also, it may be regarded as a strength that multiple detailed alcohol and tobacco use variables were utilized. Additionally, due to exceptional electronic medical records systems, there is almost complete participant retention with only a very small proportion of cohort members emigrated or by other means lost during the follow‐up. In fact, in the specific setting of Finnish medical system, loss to follow‐up typically indicates a lack in any major health care problem. A further strength introduced by the electronic medical records system is the exceptional completeness of the alcohol use data.

However, there are also limitations. Underreporting of alcohol use is a concern^33^, ^34^ and may attenuate effect size estimates. Thus not having collateral objective information of alcohol exposure provided by gold standard biochemical markers such as gamma‐glutamyl transferase (GGT) and carbohydrate‐deficient transferrin (CDT) in combination^35^ or phosphatidylethanol^36^ may be regarded as a limitation. However, alcohol use was assessed was at each hospital visit and during hospital stay, which limits underreporting‐related concerns. Moreover, a proxy measure for alcohol use disorders such as the AUDIT^37^ would have provided valuable complementary information particularly for disentangling carcinogen exposure‐related effects from other adverse effects induced by sustained alcohol use. On the other hand, previous studies utilizing solely AUDIT‐based exposure variables have reported nonsignificant findings.^17^

## 5 CONCLUSIONS

After receiving diagnosis of HNSCC, continuation of alcohol use, even at moderate level, that is, 10–20 units/week, associates with increased mortality risk, independent of age at diagnosis, tumor stage, and tobacco use status. HNSCC patients with moderate alcohol use would most probably benefit from alcohol brief interventions. Thus, implementing non‐labor‐intensive systematic screening of alcohol use and alcohol brief interventions in treatment and follow‐up of patients with HNSCC is of paramount importance.

## AUTHOR CONTRIBUTIONS

Johannes Routila had full access to all the data in the study and takes responsibility for the integrity of the data and the accuracy of the data analysis. *Concept and design*: Alexander Denissoff, Solja Niemelä, and Johannes Routila. *Acquisition, analysis, or interpretation of data*: Alexander Denissoff, Teemu Huusko, and Johannes Routila. *Drafting of the manuscript*: Alexander Denissoff. *Critical revision of the manuscript for important intellectual content*: Teemu Huusko, Sami Ventelä, Solja Niemelä, and Johannes Routila. *Statistical analysis*: Alexander Denissoff and Johannes Routila. *Obtained funding*: Alexander Denissoff and Johannes Routila. *Administrative, technical, or material support*: Solja Niemelä, Sami Ventelä, and Johannes Routila. *Supervision*: Johannes Routila.

## CONFLICT OF INTEREST

The authors declare that there is no conflict of interest that could be perceived as prejudicing the impartiality of the research reported.

## Supporting information


**Figure S1** The relationship between tobacco use and overall survival (OS). Both tobacco history exceeding (A) 20 pack years, and (B) 40 pack years had a significant association with OS. However, a more significant prognostic role was associated with (C) continued tobacco use after cancer diagnosis, and especially (D) smoking at the time of diagnosis. CI: confidence interval; HR, hazard ratio.

**Table S1** The survival effects of treatment protocols assessed in the final Model 4.

## Data Availability

The data that support the findings of this study are available from the corresponding author upon reasonable request.
