# Supplementary material for: Exposure to alcohol and overall survival in head and neck cancer: A regional cohort study
Source: Head Neck. 2022 Jun 17;44(10):2109–17. doi: 10.1002/hed.27125 (PMC9545212; doi:10.1002/hed.27125)
Supplement: Supplementary file 2 — Table S1 The survival effects of treatment protocols assessed in the final Model 4. [file HED-44-2109-s002.docx]

| **Supplemental Table 1.** The survival effects of treatment protocols assessed in the final Model 4.* | | | | | | | | |
| --- | --- | --- | --- | --- | --- | --- | --- | --- |
|  |  | **Total** | | **Survival at 5 yrs** | | | **Survival effect** | |
|  |  | *No. of patients* | *%* | *No. of patients* | *%* | *HR (95% CI)* | | *p* |
| **Treatment protocol** | |  |  |  |  |  | |  |
|  | *Surgery only* | 362 | 35 % | 253 | 70 % | 1 | | - |
|  | *Radiotherapy alone* | 85 | 8 % | 31 | 36 % | 2.07 (1.40 to 3.02) | | <0.001 |
|  | *Chemoradiotherapy alone* | 159 | 15 % | 77 | 48 % | 1.01 (0.69 to 1.47) | | 0.96 |
|  | *Radiotherapy + surgery* | 85 | 8 % | 38 | 45 % | 1.30 (0.91 to 1.87) | | 0.15 |
|  | *Chemoradiotherapy + surgery* | 287 | 28 % | 169 | 59 % | 0.94 (0.67 to 1.31) | | 0.73 |
|  | *No treatment* | 55 | 5 % | 1 | 2 % | 7.22 (4.80 to 10.9) | | <0.001 |
| *Survival effect was analyzed using a multivariable Cox proportional hazards model adjusting for patient age, high T class, nodal positivity, primary tumour site, tobacco use at the time of diagnosis, history of severe harmful alcohol use with major somatic complications, and current alcohol consumption of at least 10 units per week. Patient numbers, hazard ratios (HR), 95% confidence intervals (CI), and p values are presented. | | | | | | | | |
